# Modification of albendazole pharmacokinetics by menbutone administration in calves

**DOI:** 10.3389/fvets.2026.1834486

**Published:** 2026-05-08

**Authors:** E. Milena Vazquez, Beatriz Romero, Raquel Diez, Cristina Lopez, Jose M. Rodriguez, Nelida Fernandez, M. Jose Diez, Ana M. Sahagun

**Affiliations:** Department of Biomedical Sciences, Veterinary Faculty, Institute of Biomedicine (IBIOMED), University of Leon, Leon, Spain

**Keywords:** albendazole, albendazole sulfone, albendazole sulfoxide, bioavailability, calves, interaction, menbutone, pharmacokinetics

## Abstract

Optimizing the use of the existing anthelmintics has become a priority, as resistance is a growing concern worldwide and the development of novel antiparasitic drugs slow. The paper describes the pharmacokinetic interaction between the antiparasitic albendazole (ABZ) and the choleretic drug menbutone (MEN) in calves, when ABZ was administered orally (7.5 mg/kg) alone or with intramuscular MEN (10 mg/kg) (1 or 2 doses 24 h apart) to 12 animals. Blood samples were collected at 14 sampling points over 72 h, and plasma concentrations of ABZ, albendazole sulfoxide (ABZSO, active metabolite), and albendazole sulfone (ABZSO_2_, inactive one) were measured using high performance liquid chromatography (HPLC). Pharmacokinetic parameters were calculated with non-compartmental methods. No ABZ was detected at any sampling time, whereas ABZSO was determined for longer than ABZSO_2_. A significant increase in systemic exposure of ABZSO was observed following concomitant administration of MEN, with a mean ABZSO maximum plasma concentration (C_max_) and area under the plasma concentration-time curve (AUC) 33.5 and 31.8% higher, respectively, when a dose of MEN was administered. The second administration of MEN did not improve the amount absorbed compared with a single dose of the choleretic drug. As for ABZSO_2_, C_max_, AUC and t_max_ values were similar regardless the treatment followed (ABZ alone or with one or two doses of MEN). Thus, our results show that the simultaneous administration of ABZ with a single dose of MEN in cattle may increase the amount of ABZSO and maintain the efficacy of ABZ, whereas a second dose of MEN offers no such benefit.

## 1 Introduction

Parasites, such as gastrointestinal or lung nematodes, cestodes and liver flukes, are a cause for great concern in ruminant species, and may negatively impact the sustainability of production. In fact, helminth diseases costed the European ruminant livestock industry more than 1.8 billion € annually (1). In cattle, helminths impair growth and reproduction, reduce milk and meat production (2–4), and make it difficult to maintain health and welfare standards.

Ruminant helminth control highly depends on the use of antiparasitic drugs (1, 5). Nevertheless, after years of intensive use of the available compounds, anthelmintic resistance has been described in different countries, and may represent a serious obstacle for the control of parasites in this animal species (6–8). The emergence of anthelmintic resistance falls within the “One-Health” framework, as human activities and human relationship with nature have a significant impact on animal health, and threatens public health.

Over the last decade, resistance to anthelmintics in cattle has risen sharply worldwide, despite developing more slowly than in small ruminants and horses (7, 9–12). In addition, the high costs of drug development and the length of the assays make it unlikely that new pharmacological groups will be available in the near future (13). Thus, there is an urgent need to find novel pharmacological strategies to sustain parasite control in livestock (5).

Due to the difficulties in the development of novel anthelmintic compounds, optimizing the use of the existing drugs is a priority. Albendazole (ABZ) is one of the anthelmintic drugs commonly used in cattle, especially in dairy cows. This compound is a methylcarbamate benzimidazole effective against lungworms and gastrointestinal (GI) nematodes, tapeworms and liver flukes (14, 15). In susceptible parasites, ABZ binds to parasite ß-tubulin, inhibiting microtubules polymerization, which leads to changes in the energetic metabolism of parasites and causes paralysis and death (16). On the other hand, ABZ suffers a complex and extensive biotransformation in ruminants, with the parent drug rapidly metabolized into the active metabolite first (ABZ sulfoxide, ABZSO) and then the inactive one (albendazole sulfone, ABZSO_2_) (17).

As other benzimidazoles, ABZ is poorly soluble in water, which explains its variable and incomplete bioavailability (18). Dissolution in the gastrointestinal tract is a rate- and extent-limiting factor for ABZ (19, 20), and small differences in solubility may have significant effects on its absorption and bioavailability (21). Increasing drug bioavailability and thus, enhancing drug exposure, is a well-established pharmacological approach that may also limit the emergence of resistance. Several approaches have been employed to achieve this objective with ABZ, including alternative formulations to improve ABZ aqueous solubility (22–29), the modulation of its hepatic biotransformation (30, 31), or the use of non-chemical practices such as feed restriction or fasting before treatment (32–35). This paper describes a different approach on pharmacokinetic optimization, by concomitant administration of a drug for a different therapeutic purpose, menbutone (MEN), a choleretic agent that may improve ABZ solubility and intestinal absorption. MEN has been widely used in clinical practice to treat digestive disorders in several animal species for years, as it increases biliary, pancreatic and peptic secretions (36, 37).

In a previous study (38), a single dose of MEN improved ABZ bioavailability in sheep. Nevertheless, the Summary of Product Characteristics (SPC) of those medicinal products containing this choleretic drug describes that MEN may be administered once or twice, 24 h apart. Consequently, the goal of the current study was to characterize the *in vivo* drug-drug interaction between MEN and ABZ in cattle, and to evaluate if this choleretic agent may modify the pharmacokinetic pattern of ABZ and its two main metabolites (ABZSO and ABZSO_2_) when MEN was given concurrently once or twice.

## 2 Material and methods

### 2.1 Animals

Animals were eligible for the study if they were healthy and had no history of drug treatment the preceding 30 days. Calves were determined to be healthy based on physical examination by a veterinarian, who also assessed animals' health throughout the study. Twelve healthy female 4-month-old Holstein ruminant calves (body weight 75–102 kg) were then included in the study. They were kept at the farm of origin, situated in the province of Leon (Spain). Calves were separated from other animals of the same age group that did not participate in the trial, and maintained in a pen with straw and sawdust bedding. A 7-day acclimation period was followed prior to the initiation of the study. Once administration and sampling protocols were completed, all the animals were returned to their initial group. Calves were fed hay and straw *ad libitum*, a growing feed twice a day (daily ration 3–4 kg), and had unrestricted access to fresh water throughout the study. All procedures were performed in accordance with the European and Spanish regulations for animal experiments (39, 40), and approved in advance by the Institutional Animal Care and Use Committee at the University of Leon (OEBA-ULE-015-2023).

### 2.2 Drug administration and sampling

**Figure 1:**
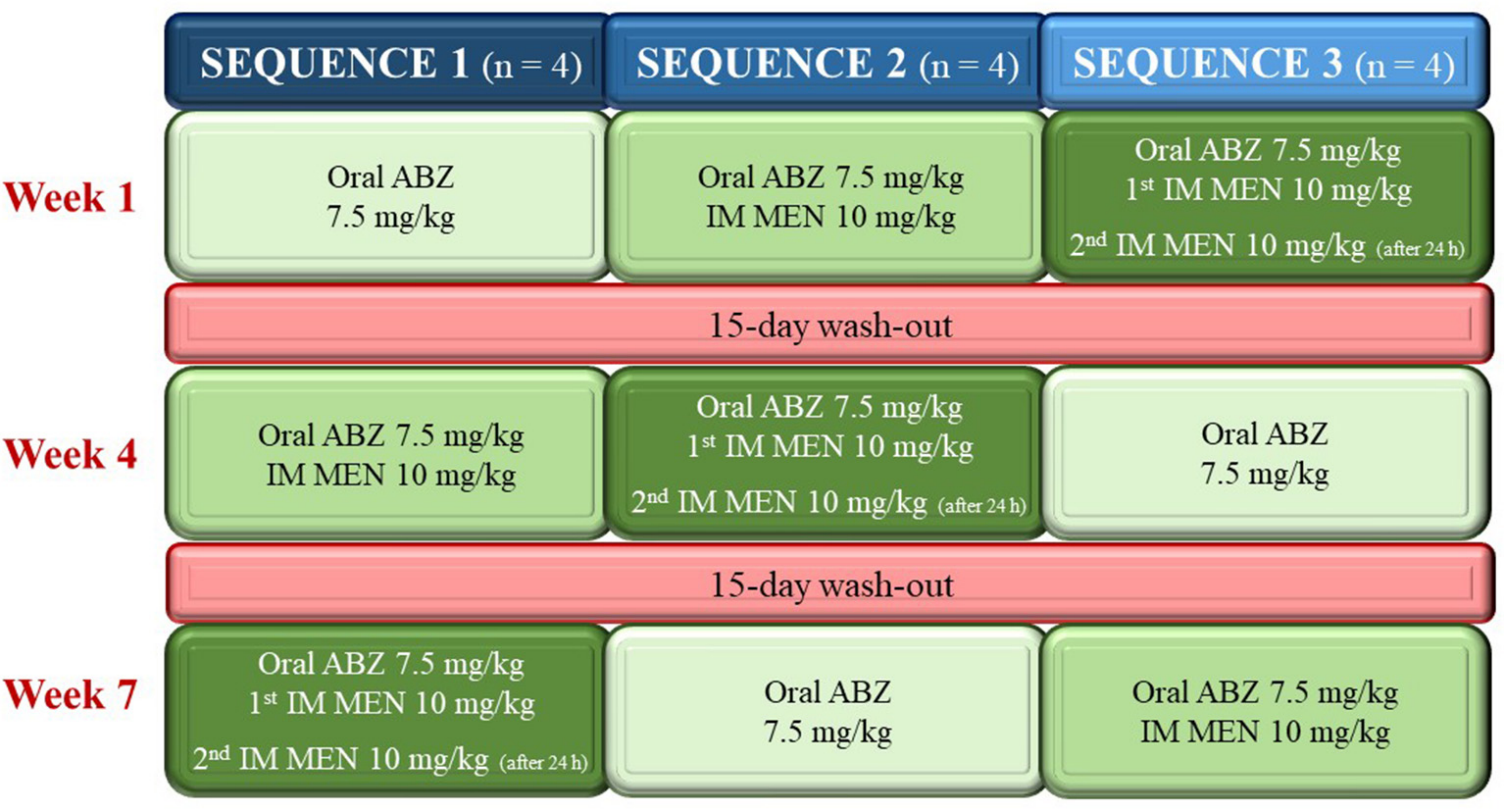
Study design.

The study was carried out under field conditions. Two commercial formulations were used to administer oral ABZ (Albendex^®^ 100 mg/mL oral suspension, S.P. Veterinaria S.A., Riudoms, Spain) and intramuscular (IM) MEN (Digestosyva^®^ 100 mg/mL solution for injection, Laboratorios Syva S.A.U., León, Spain). A three-period crossover study design was followed to evaluate the pharmacokinetic interaction between both drugs (ABZ and MEN). Animals were randomly assigned into one of the three treatment sequences (*n* = 4), which only differed in the order of treatments (Figure 1): treatment A: oral ABZ (7.5 mg/kg); treatment B: oral ABZ (7.5 mg/kg) and IM MEN (10 mg/kg, one dose); treatment C: oral ABZ (7.5 mg/kg) and IM MEN (10 mg/kg, two doses 24 h apart). Treatments were given in different order in each sequence (A/B/C; B/C/A; C/A/B). A 15-day wash-out period between treatments was always followed to minimize any possibility of carryover and ensure the complete elimination of both drugs from the animals. The drugs were administered in accordance to the SPC of the medicinal products: ABZ was administered as oral suspension with a dosing device, whereas MEN was injected intramuscularly into the neck, immediately after oral ABZ. When the animals received two doses of the choleretic drug, the first administration was made on the right side of the neck, and the second one on the left side after 24 h. Body weights were always recorded 24 h before administration to calculate appropriate dosage.

Blood was collected via venipuncture from both jugular veins alternately into 10 mL heparinized tubes (Vacutainer^®^, BD, Plymouth, UK). Blood samples were obtained immediately before dosing, and then subsequently at 0.25, 0.5, 1, 2, 3, 4, 6, 8, 12, 24, 48, 60, and 72 h. When MEN was intramuscularly administered, the first sample was always taken from the contralateral side used for drug injection. Samples underwent centrifugation at 1500 rpm for 20 min, and plasma was frozen and stored at −20 °C for a maximum of 12 weeks prior to analysis (the validated method followed would allow to storage them until 6 months) (41).

### 2.3 Drug analysis

Plasma samples were assayed for ABZ and metabolite concentrations (ABZSO and ABZSO_2_) using a high performance liquid chromatography (HPLC) method previously validated in cattle (41) according to the EMA/CHMP/ICH/172948/2019 guideline (42).

Pure reference standards (≥98% Vetranal analytical standard) of albendazole, albendazole sulfoxide, albendazole sulfone and oxibendazole (internal standard, IS) were obtained from Sigma-Aldrich (Merck, Darmstadt, Germany). HPLC and analytical grade reagents were purchased in different commercial sources: methanol (Li-Chrosolv Merck, Madrid, Spain), acetonitrile (Li-Chrosolv Merck, Madrid, Spain), ammonium acetate (VWR Chemicals, Leuven, Belgium), sodium hydroxide 1N (Panreac Quimica S.A., Barcelona, Spain), and hydrochloric acid 1M (Analyticals Carlo Erba, Milano, Italy). Purified water was produced using a Millipore Milli-Q Gradient water purification system (Waters Corporation, Mildford, MA, USA).

HPLC analyses were performed with a Waters Alliance e2695 module and a photodiode array detector (PDA) (model 2998) (Waters Corporation, Milford, MA, USA). Separation was achieved at ambient temperature on a XBridge C_18_ column (4.6 mm × 250 mm, 5 μm, Waters). A gradient elution method with 0.025 M ammonium acetate buffer (pH 6.6) as solvent A and acetonitrile as solvent B was used as follows: 0–5 min 73%−50 % A; 4 min 50 % A; 1 min, 50%−73 % A; 2 min, 73% A, with a flow rate of 1.2 mL/min. Injection volume was 50 μL, and peaks were detected at a wavelength of 292 nm. Under these conditions, time retentions were 3.5 min for ABZSO; 4.9 min for ABZSO_2_; 7.0 min for IS and 8.5 min for ABZ. Regarding MEN, no interference was noted as its detection wavelength was 236 nm and the retention time 4.5 min.

Sample processing was carried out using solid phase extraction (SPE) procedure with Oasis HLB cartridges (3 cc, 60 mg; Waters Corporation, Milford, MA, USA). Cartridges were preconditioned with 1 mL methanol and 1 mL water. Then, 1 mL plasma (spiked with 0.1 mL IS, 10 μg/mL) was applied and drawn through the cartridge. It was washed with 3 mL water and dried with air for 5 min. Elution was made with 2 mL methanol, and the solvent evaporated to dryness under a gentle flow of nitrogen. Samples were redissolved into 0.25 mL mobile phase (ammonium acetate buffer 0.025 M/acetonitrile; 73:27), and a 50 μL aliquot injected into the HPLC system.

The selectivity, lower limit of quantification (LLOQ), calibration linearity, accuracy, precision, stability and limit of detection (LOD) were established following EMA guidelines for bioanalytical analysis (42).

### 2.4 Pharmacokinetic analysis

Compartmental and non-compartmental analyses were performed for individual concentration-time profiles with Phoenix WinNonlin 8.4 (Certara, NJ, USA). One- and two- compartment open models with 1/C weighting were assessed, and the best fit chosen according to Akaike information criterion, the residuals and the coefficient of variations obtained (43–46). Non-compartmental approach was based on the statistical moments theory and traditional formulae (46–49). Key pharmacokinetic parameters were calculated, including the elimination rate constant (λ), terminal elimination half-life (*t*_1_/__2_λ_), area under the curve (AUC), area under the moment curve (AUMC), and mean residence time (MRT). λ was calculated by least squares regression of the logarithm of plasma concentration-time data over the terminal elimination phase, and *t*_1_/__2_λ_ as 0.693/λ. AUC and AUMC were calculated to the last measurable concentration using the linear trapezoidal method, and then extrapolated to infinity. MRT was calculated as AUMC/AUC, and C_max_ and *t*_max_ were ascertained by visual examination of data.

### 2.5 Statistical analysis

Statistical analyses were performed by IBM SPSS software v. 26 (IBM Corporation, Armonk, NY, USA). Pharmacokinetic parameters were expressed as mean ± standard deviation (SD). The Shapiro-Wilk test was used to check if data exhibited a normal distribution. When normal, repeated measures ANOVA and *t* test were used. If not, the non-parametric Friedman and Wilcoxon tests were then employed. To assess the influence of the crossover design the repeated measures ANOVA test was used. A *p* value ≤ 0.05 was considered significant in all statistical analyses.

## 3 Results

As mentioned above, ABZ, ABZSO and ABZSO_2_ were determined by HPLC following a method validated previously (41), no interfering peaks were detected in the blank samples at the retention times of the three analytes (ABZ, ABZSO, and ABZSO_2_). Linear regression calibration curves (0.025 to 2.0 μg/mL) were calculated by plotting the peak area ratio of each analyte to the IS vs. nominal concentration, with correlation coefficients (R^2^) ≥0.999. LLOQ was 0.025 μg/mL for ABZ and metabolites (ABZSO and ABZSO_2_). Recovery was 101.6 ± 0.7 %, 100.4 ± 1.3 % and 100.0 ± 1.4 % for ABZ, ABZSO, and ABZSO_2_, respectively. Both intra- and inter-day accuracy and precision, as well as stability for all temperatures and times evaluated fulfilled the requirements of the EMA/CHMP/ICH/172948/2019 guideline (42).

**Figure 2:**
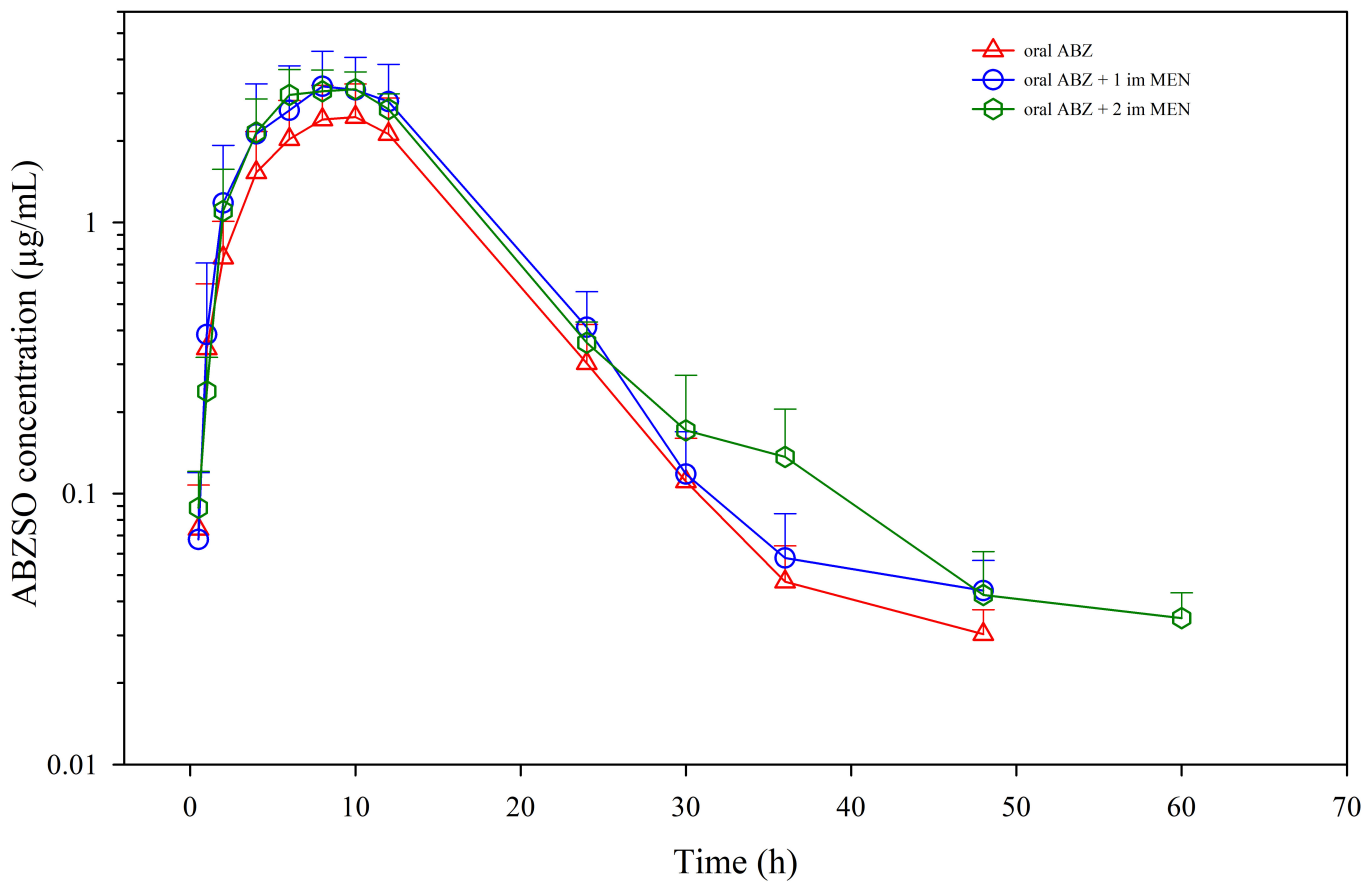
Plasma concentrations (mean ± SD) of ABZSO obtained after oral ABZ administration (7.5 mg/kg), oral ABZ (7.5 mg/kg) + 1 dose intramuscular MEN (10 mg/kg), and oral ABZ + 2 doses intramuscular MEN (10 mg/kg) 24 h apart to 12 calves.

**Figure 3:**
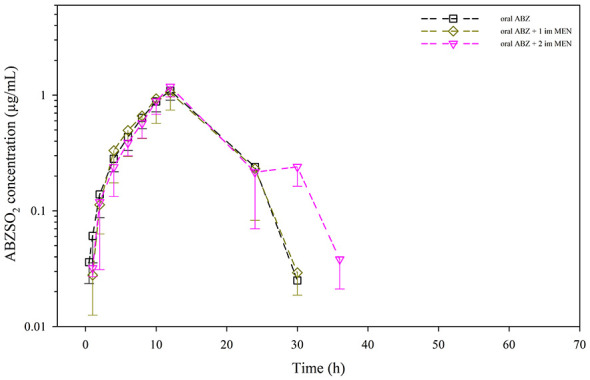
Plasma concentrations (mean ± SD) of ABZSO_2_ obtained after oral ABZ administration (7.5 mg/kg), oral ABZ (7.5 mg/kg) + 1 dose intramuscular MEN (10 mg/kg), and oral ABZ + 2 doses intramuscular MEN (10 mg/kg) 24 h apart to 12 calves.

No adverse events were observed in any of the animals treated, showing good tolerability when ABZ was used alone or with MEN. Regarding drug determination, the parent drug (ABZ) was not detected in any plasma sample obtained after treatments, which reflects its rapid conversion to ABZSO. Mean and individual plasma concentrations of ABZSO and ABZSO_2_ throughout the sampling period for the three treatments are shown in Figures 2, 3, and Supplementary Figure S1. ABZSO (active metabolite) was present in plasma at higher concentrations and for a longer time than ABZSO_2_ (inactive metabolite): ABZSO was detected from 0.5 to 48 h after having administered ABZ alone or ABZ + MEN (1 dose), and up to 60 h when animals received ABZ and 2 doses of the choleretic agent (Figure 2). As for ABZSO_2_, plasma concentration pattern was slightly different, as it was detected between 0.5 and 30 h post-administration when only ABZ was administered, whereas co-administration of MEN delayed its appearance to 1 h post-administration (Figure 3). When MEN was administered twice, ABZSO_2_ was present longer (36 h) than with ABZ alone or with only one dose of MEN (30 h). Plasma concentration profiles tend to be similar for both metabolites regardless the treatment followed. However, some differences have been observed for ABZSO, as higher concentrations were obtained with MEN administrations. Moreover, when two doses of MEN were administered concurrently, ABZSO concentrations increased at the last sampling times, as shown in mean and most individual plasma concentration-time profiles. These differences are not so marked for ABZSO_2_, as concentrations tend to be slightly higher when a single dose of MEN was injected, and only at 30 h ABZSO_2_ concentrations were clearly higher with two doses of MEN.

Concentration-time data of ABZSO or ABZSO_2_ did not properly fit compartmental models. Thus, only non-compartmental parameters were calculated. Table 1 shows the mean non compartmental pharmacokinetic parameters for ABZSO. C_max_ increased significantly when the anthelmintic was co-administered with MEN (2.57 μg/mL with ABZ alone vs. 3.43 μg/mL with ABZ + 1 dose MEN; and 3.44 μg/mL with ABZ + 2 doses MEN). The same behavior was observed for AUC_last_, with significantly higher values when the choleretic was administered compared to administration of ABZ alone, although the second administration of MEN 24 h apart did not provide any advantage over injecting MEN only once. As for *t*_max_, concomitant administration of MEN did not significantly change absorption rate (9.0 h with ABZ alone; 9.3 h with ABZ + MEN 1 dose, and 8.5 h with ABZ + MEN 2 doses). On the other hand, the second administration of MEN increased significantly ABZSO elimination half-life (5.16 h with ABZ alone; 5.03 h with ABZ + MEN 1 dose; and 8.74 h with ABZ + MEN 2 doses), which may be associated with higher and more prolonged concentrations at the last sampling points, as described before. This behavior was observed when individual parameters were assessed. When treatment with ABZ alone was compared with ABZ-MEN (1 dose), C_max_ increased in 8 calves, did not change in 2 animals and was lower in 2 calves, whereas AUC_last_ raised in 9 animals and diminished in 3. However, if comparisons were made between both concomitant treatments of MEN (1 or 2 doses), ABZSO C_max_ increased in 5 animals, decreased in 6 and did not vary in 1 calf, and something similar occurred with AUC_last_, as raised in 6 calves, did not change in 1 and reduced in 5 animals. No significant differences among the same treatments of the crossover sequences carried out were revealed (repeated measures ANOVA).

**Table 1:** Non-compartmental pharmacokinetic parameters (mean ± SD) of albendazole sulfoxide (ABZSO) obtained after oral ABZ administration (7.5 mg/kg), oral ABZ (7.5 mg/kg) + 1 dose intramuscular MEN (10 mg/kg), and ABZ oral (7.5 mg/kg) + 2 doses intramuscular MEN (10 mg/kg) 24 h apart to 12 calves.

Pharmacokinetic parameter	Unit	ABZ	ABZ + 1MEN	ABZ + 2MEN
λ^b, c^	h^−1^	0.137 ± 0.020	0.148 ± 0.036	0.087 ± 0.026
*t*_1/2λ_^e, f^	h	5.16 ± 0.71	5.03 ± 1.53	8.74 ± 3.10
C_max_^a, b^	μg/mL	2.57 ± 0.84	3.43 ± 1.05	3.44 ± 0.60
*t*_max_	h	9.0 ± 2.8	9.3 ± 1.6	8.5 ± 1.7
V_z_/F^f^	L/kg	1.70 ± 0.81	1.53 ± 1.70	1.94 ± 0.69
Cl/F^d^	mL/kg/h	222.6 ± 85.5	183.1 ± 114.5	154.5 ± 18.6
AUC_last_^a, b^	μg·h/mL	36.8 ± 10.3	48.5 ± 16.5	48.7 ± 5.4
${\text{AUC}}_{0-\infty }^{\text{a}}$	μg·h/mL	37.1 ± 10.4	48.8 ± 16.4	49.1 ± 5.4
AUMC_last_^a^	μg·h^2^/mL	403.9 ± 106.3	527.3 ± 147.5	561.1 ± 63.7
${\text{AUMC}}_{0-\infty }^{\text{d},\text{e}}$	μg·h^2^/mL	417.2 ± 107.7	544.3 ± 141.6	592.9 ± 72.9
MRT_last_	h	11.0 ± 1.1	11.2 ± 1.4	11.6 ± 1.0
MRT_0−∞_	h	11.3 ± 1.2	11.6 ± 1.8	12.1 ± 1.1

λ, slope of terminal phase; t_1/2λ_, half-life associated with λ; C_*max*_, maximum plasma concentration; t_*max*_, time to reach C_*max*_; V_*z*_/F, apparent volume of distribution; Cl/F, apparent clearance; AUC_*last*_, area under the plasma concentration-time curve from cero to last collected time point; AUC_0−∞_, area under the plasma concentration-time curve from cero to infinity; AUMC, area under the first moment curve; MRT, mean residence time.

Significant differences between^*a*^ABZ and ABZ + 1MEN;^*b*^ABZ and ABZ + 2MEN; and^*c*^ABZ + 1MEN and ABZ + 2MEN (t test p ≤ 0.05);^*d*^ABZ and ABZ + 1MEN;^*e*^ABZ and ABZ + 2MEN; and^*f*^ABZ + 1MEN and ABZ + 2MEN (Wilcoxon test p ≤ 0.05).

As for the inactive metabolite (ABZSO_2_), plasma concentration profiles were similar regardless whether the parent drug (ABZ) was administered with MEN (1 or 2 doses) or without this compound (Figure 3). Differences in plasma concentrations after treatments are not so marked as for ABZSO, with ABZSO_2_ concentrations slightly higher when a single dose of MEN was injected, and only at sampling time 30 h (6 h after the second administration of MEN) concentrations were clearly higher when MEN was administered twice, even higher than those of ABZSO. In fact, 8 animals showed a second peak of ABZSO_2_ at 30 h. Table 2 summarized non-compartmental pharmacokinetic parameters of ABZSO_2_. No significant differences were revealed for either C_max_ (1.10–1.19 μg/mL), AUC_last_ (14.6–16.2 μg·h/mL) or t_max_ (11.2–12.0 h) regardless the treatment followed. Again, an increase in *t*_1/2λ_ was observed with the number of MEN doses administered (3.75 h with ABZ alone; 4.18 h with ABZ + MEN 1 dose, and 5.38 h with ABZ + MEN 2 doses).

**Table 2:** Non-compartmental pharmacokinetic parameters (mean ± SD) of albendazole sulfone (ABZSO_2_) obtained after oral ABZ administration (7.5 mg/kg), oral ABZ (7.5 mg/kg) + 1 dose intramuscular MEN (10 mg/kg), and ABZ oral (7.5 mg/kg) + 2 doses intramuscular MEN (10 mg/kg) 24 h apart to 12 calves.

Pharmacokinetic parameter	Unit	ABZ	ABZ + 1MEN	ABZ + 2MEN
λ^a, b^	h^−1^	0.191 ± 0.034	0.174 ± 0.035	0.131 ± 0.015
*t*_1/2λ_^a, b^	h	3.75 ± 0.72	4.18 ± 1.03	5.38 ± 0.63
C_max_	μg/mL	1.12 ± 0.16	1.10 ± 0.34	1.19 ± 0.22
*t*_max_	h	11.8 ± 0.6	11.2 ± 1.3	12.0 -
V_z_/F^a^	L/kg	2.78 ± 0.467	3.19 ± 1.20	3.64 ± 0.80
Cl/F	mL/kg/h	518.6 ± 74.4	519.9 ± 110.2	469.0 ± 89.7
AUC_last_	μg·h/mL	14.6 ± 2.0	14.9 ± 3.3	16.2 ± 2.9
AUC_0−∞_	μg·h/mL	14.7 ± 2.0	15.0 ± 3.2	16.5 ± 3.0
${\text{AUMC}}_{\text{last}}^{\text{a}}$	μg·h^2^/mL	181.2 ± 49.5	186.1 ± 44.4	227.4 ± 50.8
${\text{AUMC}}_{0-\text{∞}}^{\text{a}}$	μg·h^2^/mL	185.3 ± 50.6	192.3 ± 42.7	240.9 ± 54.5
MRT_last_^c, d^	h	12.3 ± 2.0	12.5 ± 1.7	14.0 ± 1.2
${\text{MRT}}_{0-\text{∞}}^{\text{c},\text{d}}$	h	12.4 ± 2.0	12.9 ± 1.7	14.5 ± 1.3

λ, slope of terminal phase; t_1/2λ_, half-life associated with λ; C_*max*_, maximum plasma concentration; t_*max*_, time to reach C_*max*_; V_*z*_/F, apparent volume of distribution; Cl/F, apparent clearance; AUC_*last*_, area under the plasma concentration-time curve from cero to last collected time point; AUC_0−∞_, area under the plasma concentration-time curve from cero to infinity; AUMC, area under the first moment curve; MRT, mean residence time.

Significant differences between^*a*^ABZ and ABZ + 2MEN; and^*b*^ABZ + 1MEN and ABZ + 2MEN (t test p ≤ 0.05);^*c*^ABZ and ABZ + 2MEN; and^*d*^ABZ + 1MEN and ABZ + 2MEN (Wilcoxon test p ≤ 0.05).

## 4 Discussion

As previously reported in cattle (28, 32, 33, 35, 50, 51) and other ruminants (38, 52–55), the parent compound (ABZ) was undetectable in plasma at any sampling time in the current study due to its fast sulfoxidation in the gastrointestinal tract and liver. Thus, ABZSO and ABZSO_2_ were the only compounds determined in plasma after oral ABZ administration, regardless of the treatment followed (ABZ alone or with MEN). ABZSO was the main metabolite recovered in plasma for 48–60 h in calves, with higher and longer-lasting concentrations than the inactive metabolite (ABZSO_2_).

Although the goal of this study was to evaluate the interaction between ABZ and MEN, we will first discuss the pharmacokinetics of ABZ. In this sense, limited information is available on the pharmacokinetics of ABZ after oral administration in cattle. In fact, we have found no pharmacokinetic study in which the parent drug (ABZ) has been administered by this route to this animal species at the same dose as in our study (7.5 mg/kg), and only two in which the doses used were 5 mg/kg (56) and 10 mg/kg (57), respectively. A few other authors described ABZ pharmacokinetics in cattle after intraruminal administration (28, 32, 33, 35, 50, 51). Consequently, comparisons have only been made with the few studies available on cattle (all of them conducted on calves) and those carried out in the other two main ruminant species (sheep and goats), in this case with the same oral dose as that used in this study (7.5 mg/kg) (31, 54, 57–60). In our study, as explained before, the anthelmintic was orally administered under field conditions, and according to the SPC of the commercial product used. Alvarez et al. (61) described that C_max_ and AUC values for ABZSO did not increase proportionally with doses, and something similar has been observed in cross-bred cattle (50).

After ABZ administration, mean ABZSO C_max_ was higher than those previously reported with oral doses of 5 mg/kg (0.57 μg/mL) (56) and 10 mg/kg (0.75 μg/mL) (57), as well as intraruminal ones ranging from 7.5 to 10 mg/kg (0.43-2.18 μg/mL) (28, 32, 33, 35, 50, 51, 56) whereas a higher C_max_ was achieved (6.52 μg/mL) with 15 mg/kg (50). AUC_last_ was higher than that calculated with the oral dose of 5 mg/kg (9.8 μg·h/mL) (56), and between those values established after intraruminal administration (31.2–38.4 μg·h/mL) at 7.5 mg/kg (50, 51). Lower C_max_ values were reported in sheep (1.30–2.38 μg/mL) (54, 58, 59) and goats (0.36–2.38 μg/mL) (31, 57, 60) after oral administration of 7.5 mg/kg. Only Lifschitz et al. (58) reported a C_max_ slightly higher (2.7 μg/mL) than ours in fasted sheep.

Regarding *t*_max_, the values observed in the current study were similar to that indicated after oral administration (9.71 h) (56), and shorter than those reported by other authors in cattle when ABZ was administered intraruminally (11.2–19 h) (28, 32, 33, 35, 50, 51). When compared with other ruminant species, our t_max_ values are intermediate between those indicated in sheep (7–10.6 h) (54, 58, 59) and shorter than in goats (11.43–19 h) (31, 57, 60). As for MRT_last_, mean values in this study are between those indicated previously in the same animal species with intraruminal administration (9.63–16.82 h) (28, 32, 33, 35), and shorter than those calculated in sheep and goats orally (14–21.9 h) (31, 58, 59). These variations may be attributed to the different ways of ABZ administration, the commercial products used, the age of the animals or interspecies differences. In this sense, metabolic differences for ABZ have been described between sheep and cattle (62), which may account for the shorter residence times of ABZSO described in cattle.

We have observed that concurrent MEN administration substantially increased the amount absorbed of ABZSO (C_max_ and AUC) without affecting the rate of absorption (*t*_max_). This increase accounts for 33.5 % in C_max_ and 31.8 % in AUC_last_ when only one dose of MEN was intramuscularly injected. In the case of AUC_last_, the administration of the second dose of MEN 24 h later did not significantly improve this parameter (32.3 %) compared to the administration of a single dose of the choleretic agent. The higher amounts in plasma of ABZSO after MEN administrations may be related to improved ABZ solubilization and absorption rather than delayed elimination. ABZSO plasma concentration pattern changed not only with the first administration of the choleretic drug but also with the second one, with higher and prolonged concentrations of the active derivative in the last sampling times. Significantly higher values have been calculated for *t*_1/2λ_ with the second dose of MEN, although MRT values did not change with treatments (ABZ alone or with MEN; no significant differences).

In a previous study carried out in sheep (38), intramuscular MEN administration (10 mg/kg, one dose) with oral ABZ (5 mg/kg) led to significant increases in ABZSO C_max_ (12.8 %) and AUC_last_ (21.5 %), with increments smaller than those observed in the current study. These disparities may be related to several factors such as interspecies differences or the commercial products used. Regarding the medicinal products, the same product containing MEN was used as in sheep (38), but in the case of ABZ a different approved commercial medicine indicated for cattle was employed in the current study. The age of the animals is another factor to be considered, as in the study of Diez et al. (38) sheep were adult ones and in our assay the animals, although ruminant, were younger. Age is an important physiological factor that may alter the pharmacokinetics of drugs, but little is known in veterinary medicine about its effect in cattle and other animal species. Cattle undergo crucial changes in body structure and composition as they transition from pre-ruminant to ruminant, which can significantly affect pharmacokinetic processes (63), as described for flunixin (64), danofloxacin and tulatromycin (65, 66) or marbofloxacin (67). On the other hand, inter-individual variability has also been observed in plasma concentrations and pharmacokinetic parameters among animals. Differences in the dissolution and absorption rates of albendazole, related to variations among animals in terms of ruminal content and abomasal pH, as well as the complex metabolism of this anthelmintic drug in ruminants may have influenced the results obtained. When ABZ was administered with MEN, differences in bile secretion among calves when the choleretic drug was once or twice administered may have also contributed to this variability.

A reversible exchange between blood and gastrointestinal tract has been described for benzimidazole compounds and their metabolites (68, 69). For these drugs, plasma concentrations of the active compounds (ABZSO in our study) reflect the exposure pattern of parasites located in the gastrointestinal mucosa/lumen as well as in other tissues such as lungs (32, 58, 70), and are good indicators of their clinical efficacy (32). According to the results of this study, it is clear that MEN has importantly improved plasma concentrations of ABZSO in cattle and may have a positive influence on its efficacy in this animal species, in which ABZ is commonly used as an anthelmintic, especially in dairy animals. The enhanced plasma concentrations and availability of ABZSO achieved with MEN suggest a more intense drug exposure at the parasite target sites, and may have a significant impact in anthelmintic therapy. Due to its choleretic activity, the subsequent increase of bile secretion may have accounted for greater dissolution and absorption from the small intestine due to its surfactant action. On the other hand, since ABZSO undergoes enterohepatic recirculation, the choleretic agent would also contribute to higher and sustained intestinal and plasma concentrations of the active metabolite. Higher plasma concentrations of ABZSO also mean that a greater amount of this metabolite is available to reach from the blood the gastrointestinal tract and then be reduced to ABZ.

The surfactant effect of bile salts has been also described in humans (71, 72). lt should be taken into account that ABZ bioavailability and efficacy is conditioned by its solubility (19, 20). This effect is even more evident when animals received the second administration of MEN 24 h apart the first one, increasing ABZSO plasma concentrations between 30 and 48 h post-administration and prolonging their presence in plasma up to 60 h. After intramuscular administration (10 mg/kg), MEN *t*_max_ was achieved in cattle at 1.63 h and its MRT_last_ was 6.32 h (73). Moreover, MEN intravenous administration to steers (10 mg/kg) increased bile flow 4.5-fold for at least 6 h (37). Therefore, it would be feasible that ABZSO concentrations would be affected by MEN in the last sampling times of the study, and remained higher and for longer when a second injection of the choleretic agent was administered after 24 h.

As for ABZSO_2_, information on its pharmacokinetic parameters is substantially more limited than that for ABZSO, probably because is an inactive derivative. Concentrations of this metabolite were always lower than those of the active one in the current study. ABZSO_2_ appeared rapidly after the administration of ABZ alone (0.5–1 h, depending on the animal considered) and also disappeared fast (detection was achieved only up to 30 h), whereas in sheep (38) this inactive metabolite was detected over a longer period of time (2–60 h). The rapid detection of ABZSO_2_ in cattle may be related to the greater sulphonation capacity described *in vitro* for this animal species (74). Mean ABZSO_2_ C_max_ (1.6 μg/mL) and AUC_last_ (30.4–31.2 μg.h/mL) were higher and t_max_ occurred later (18.7–20.1 h) in cross-breed cattle when the parent drug (ABZ) was intraruminally administered at the same dose (7.5 mg/kg) (50, 51). On the other hand, a great disparity was found for C_max_ (0.96–3.85 μg/mL) and t_max_ (18–26 h) when ABZ was administered to cattle at a dose of 10 mg/kg (28, 33, 35, 50, 57). A lower AUC_last_ was calculated with this latter dose (10 mg/kg) (26.3 μg·h/mL) (35). Regarding MRT, our mean values for this metabolite were somewhat lower or between those described in cattle (13.1–20.6 h) (28, 33, 35).

Concomitant administration of MEN tended to delay a little the appearance of ABZSO_2_ in plasma (up to sampling times 1 or 2 h) and, when the second dose of the choleretic agent was injected, prolonged its detection 6 h (up to 36 h). The higher concentrations of this inactive metabolite in the last two sampling points when it was detected (30 and 36 h) may in turn reflect the modifications in the concentrations of the active metabolite at those sampling times, favoring its transformation into the inactive ABZSO_2_. On the other hand, co-administration of MEN (one or two doses) did not significantly affect the pharmacokinetic behavior of the inactive metabolite, either in terms of amount (C_max_ and AUC_last_) or rate (t_max_).

Different formulation strategies have been evaluated to improve ABZ aqueous solubility, including the incorporation of surfactants (28, 75), the development of self-microemulsification formulations (76) or lipid nanoparticles (77), solid dispersions (24), or cyclodextrin complexes (78). Of those, only Virkel et al. (28) assessed the effect of surfactants in cattle, with higher plasma availability of ABZSO with sodium lauryl sulfate but not with sodium taurocholate. The commercial formulation used to administer ABZ already included surfactant agents to improve the permeability of biological membranes and increase ABZ dissolution, but concurrent administration of MEN clearly enhanced the absorption of this anthelmintic drug.

The results of this study have shown that MEN administration leads to a meaningful improvement in ABZSO exposure in calves, with significant increases in C_max_ and AUC_last_ parameters. Therefore, MEN and ABZ co-administration could have a positive impact on ABZ efficacy in cattle, particularly in dairy animals, where albendazole is commonly used, and may help to optimize nematode control in this animal species.

Drug associations are commonly used in veterinary clinical practice and have proven successful in several fields of veterinary medicine. However, information on drug interactions in cattle is quite limited. As described in this study, modification of the pharmacokinetic processes for a certain compound through a drug-drug interaction is an interesting option to improve the efficacy of ABZ in this animal species. The results presented here highlight the need for further research on drug-drug interactions involving anthelmintics, to identify the advantages or disadvantages in clinical practice of drug co-administration against helminths in livestock. The current study, carried out under field conditions, provide novel pharmacokinetic data for ABZ when used in combination with MEN, and may contribute to achieve optimal parasite control, avoid selection for drug resistance, and a more efficient use of ABZ in cattle.

## 5 Conclusion

In summary, the study carried out has demonstrated that co-administration of ABZ with a dose of MEN to cattle results in marked changes in ABZSO concentrations, increasing the amount of the active metabolite incorporated into plasma in a feasible, safe and fairly economical manner to maintain the therapeutic potential of ABZ. A second dose of this choleretic drug, although possible according to the SPC of those commercial products containing MEN, offers no advantage to practitioners.

## Data Availability

The raw data supporting the conclusions of this article will be made available by the authors, without undue reservation.
