# Supplementary material for: Modification of albendazole pharmacokinetics by menbutone administration in calves
Source: Front Vet Sci. 2026 May 8;13:1834486. doi: 10.3389/fvets.2026.1834486 (PMC13193926; doi:10.3389/fvets.2026.1834486)
Supplement: Supplementary file 1 [file Supplementary_file_1.docx]

Supplementary Material

**FIGURE S1.** Individual plasma concentrations of ABZSO and ABZSO_2_ obtained after administration of ABZ to 12 calves alone (oral, 7.5 mg/kg) or with MEN (intramuscular, 1 or 2 doses, 10 mg/kg). (**△**) ABZSO after ABZ administration alone; (⭘) ABZSO after ABZ + 1 dose MEN administration; ( ) ABZSO after ABZ + 2 doses MEN administration 24 h apart; (⬜)ABZSO_2_ after ABZ administration alone; (◇) ABZSO_2_ after ABZ + 1 dose MEN administration; and (▽)ABZSO_2_ after ABZ + 2 doses MEN administration 24 h apart.
